# Editorial: How do we see? Morphology and physiology of retinal neurons

**DOI:** 10.3389/fnana.2022.1099583

**Published:** 2023-01-16

**Authors:** Luis Perez de Sevilla, Laura Fernández-Sánchez

**Affiliations:** ^1^Department of Neurobiology, David Geffen School of Medicine at UCLA, University of California, Los Angeles, Los Angeles, CA, United States; ^2^Department of Optics, Pharmacology and Anatomy, University of Alicante, Alicante, Spain

**Keywords:** retina, CNS, ganglion cells, bipolar cells, amacrine cell, inflammation

The retina is a highly specialized tissue which lines the inner surface of the eye. As a projection of the central nervous system, and being part of the brain, the retina shares many of the characteristic traits of neuronal tissue. This makes the retina suitable not only for the study of retinal diseases and physiology, but also for neuroscience studies. The retina is located inside the eye, which is almost completely transparent, making the retina like a “window to the brain”.

The retina exhibits a highly organized structure, and the tissue is easily obtained and widely available. A wide range of invasive and non-invasive methods can be utilized to evaluate its morphology and functionality. Thus, it is an excellent option to investigate many neuronal processes to yield novel findings which can then be extrapolated to the brain.

It is well-established that many of the underlying mechanisms described in retinal diseases such oxidative stress, inflammation, neuronal development, aging and activation of programmed cell death mechanisms, are common features in both retinal and brain circuitries. The study of these mechanisms in the retina are crucial to obtaining a better understanding of brain research.

The special Research Topic entitled “*How Do We See? Morphology and Physiology of Retinal Neurons”* covers a variety of research in the retina field (from basic research to computational biology and drug experimentation). This Research Topic consists of seven original articles.

Characterizing potential changes in retinal morphology due to aging is a mandatory task in order to identify the underlying mechanisms and possible therapeutic targets in many neurodegenerative diseases. The physiological aging process of the retina is accompanied by various and sometimes extensive changes in diseases like macular degeneration, retinopathies and glaucoma. Haverkamp, Reinhard et al. aimed to characterize potential aging-associated retinal changes in the common marmoset. This study provides the first descriptions of age-associated changes in quantities, architecture, patterns and decline of different retinal cell populations in this animal model.

Relatively little is known about the role of *Sox5* in neural development. Kulesh at al. focused on the role of the transcription factor *Sox5* to control the morphological differentiation of a specific type of cone bipolar cell, the type 7 cell. They used a conditional knockout mouse line as well as the *Gustducin-gfp* reporter which labels type 7 bipolar cells to study the regulation of the differentiation during development. Bipolar cells lacking *Sox5* developed the characteristic morphological features of type 7 cells, yet their axonal and dendritic field areas were reduced in size, while the axonal arbors exhibited sprouting of vertically oriented processes. These morphological changes would have a significant impact on their specific functions affecting the visual processing.

Potassium (K^+^) channels play important roles in all cell types underlying both normal and pathophysiological functions like nerve impulse propagation, muscle contraction, cellular activation and the secretion of biologically active molecules. In addition, various K^+^ channels are recognized as potential therapeutic targets in the treatment of multiple sclerosis, Alzheimer's disease, Parkinson's disease, epilepsy, stroke, brain tumors, and many other diseases. The paper by Ogata et al. identifies an additional K^+^ channel subunit in optic nerve and provides evidence that the ion channels it forms *in situ* are poised to be modulated by lighting conditions that alter CaMKII activation in retinal ganglion cell somata and axons.

Another study by Haverkamp, Mietsch et al. provides a better understanding of structural retinal layering including morphological differences and distinctive features in the common marmoset retina. The authors used immunohistochemical approaches to immunolabel different groups of retinal neurons and described for the first time developmental retinal errors over a wide age range in the common marmoset retinas, which can be considered for future studies in this and other animal species.

Inherited retinal diseases exhibit a strong inflammatory component as well as deep remodeling affecting the vascular network. Traditionally, retinal or brain inflammation has been linked mainly to microglia, but the most recent studies indicate that macroglia, astrocytes and even Müller cells may be modulating these responses. The paper presented by Fernandez-Sanchez et al. shows the effect of TUDCA (Tauroursodeoxycholic acid) on delaying vascular network degeneration and modulating astrocyte and Müller cells changes associated with retinitis pigmentosa.

The paper by Rezeanu et al. presents a computational model that demonstrates how the cortex could use unsupervised learning to efficiently separate the signals from specific ganglion cells (the L vs M midget ganglion cells), into distinct signals for black and white based only on correlation of activity over time, and why it is unlikely that these same ganglion cells could simultaneously mediate our perception of red and green.

The paper by Gallego-Ortega et al. identifies and characterizes numerically and topographically the population of alpha retinal ganglion cells and their subtypes with a combination of specific antibodies in the adult pigmented mouse retina. Alpha retinal ganglion cells are distributed throughout the retina with a higher density in the temporal area. This study also shows that the sustained ON and OFF response subtypes are mainly located in the periphery while the transient ON and OFF response subtypes are found in the central regions of the retina.

In summary, this Research Topic for Frontiers in Neuroanatomy presented an overview of the current advances in retina research and ongoing research directions. In a variety of papers discussed above, we have emphasized the importance of this area of research and how the retina is an excellent model by which to advance our understanding of the central nervous system pathways.

## Author contributions

Editorial was written by LP and LF-S. Both authors contributed to the article and approved the submitted version.

